# Associations among unit leadership and unit climates for implementation in acute care: a cross-sectional study

**DOI:** 10.1186/s13012-018-0753-6

**Published:** 2018-04-25

**Authors:** Clayton J. Shuman, Xuefeng Liu, Michelle L. Aebersold, Dana Tschannen, Jane Banaszak-Holl, Marita G. Titler

**Affiliations:** 1 School of Nursing, University of Michigan, 400 N. Ingalls, Room 4162, Ann Arbor, MI 48109 USA; 2 School of Public Health, University of Michigan, 1415 Washington Heights, Ann Arbor, MI 48109 USA; 3 Institute of Gerontology at Michigan Medicine, 300 N. Ingalls, Ann Arbor, MI 48109 USA

**Keywords:** Implementation, Climate, Nurse manager, Evidence-based practice, Acute care, Middle manager

## Abstract

**Background:**

Nurse managers have a pivotal role in fostering unit climates supportive of implementing evidence-based practices (EBPs) in care delivery. EBP leadership behaviors and competencies of nurse managers and their impact on practice climates are widely overlooked in implementation science. The purpose of this study was to examine the contributions of nurse manager EBP leadership behaviors and nurse manager EBP competencies in explaining unit climates for EBP implementation in adult medical-surgical units.

**Methods:**

A multi-site, multi-unit cross-sectional research design was used to recruit the sample of 24 nurse managers and 553 randomly selected staff nurses from 24 adult medical-surgical units from 7 acute care hospitals in the Northeast and Midwestern USA. Staff nurse perceptions of nurse manager EBP leadership behaviors and unit climates for EBP implementation were measured using the Implementation Leadership Scale and Implementation Climate Scale, respectively. EBP competencies of nurse managers were measured using the Nurse Manager EBP Competency Scale. Participants were emailed a link to an electronic questionnaire and asked to respond within 1 month. The contributions of nurse manager EBP leadership behaviors and competencies in explaining unit climates for EBP implementation were estimated using mixed-effects models controlling for nurse education and years of experience on current unit and accounting for the variability across hospitals and units. Significance level was set at *α* < .05.

**Results:**

Two hundred sixty-four staff nurses and 22 nurse managers were included in the final sample, representing 22 units in 7 hospitals. Nurse manager EBP leadership behaviors (*p* < .001) and EBP competency (*p* = .008) explained 52.4% of marginal variance in unit climate for EBP implementation. Leadership behaviors uniquely explained 45.2% variance. The variance accounted for by the random intercepts for hospitals and units (*p* < .001) and years of nursing experience in current unit (*p* < .05) were significant but level of nursing education was not.

**Conclusion:**

Nurse managers are significantly related to unit climates for EBP implementation primarily through their leadership behaviors. Future implementation studies should consider the leadership of nurse managers in creating climates supportive of EBP implementation.

**Electronic supplementary material:**

The online version of this article (10.1186/s13012-018-0753-6) contains supplementary material, which is available to authorized users.

## Background

Middle managers, including nurse managers, are argued to influence implementation of evidence-based practices (EBPs) [1, 2]. As “links” between executive leadership and clinicians delivering care, nurse managers are ideally situated within healthcare organizations to influence implementation of EBP by leading implementation efforts and fostering a climate supportive of EBP [1–3]. Nurse managers are responsible for supervision of their unit(s), including staffing, maintaining budgets, ensuring excellent nursing practice, facilitating quality improvement, and promoting patient safety. In light of these responsibilities, the tasks of facilitating EBP integration and fostering an EBP unit climate are highly influenced by the leadership of nurse managers. Yet, there is a paucity of research on the influence of nurse managers on EBP implementation [4–6].

Leadership support for EBP is believed to be associated with EBP use by nursing staff [4, 5, 7, 8]. Based on staff nurse perceptions, previous nursing studies suggest that positive managerial leadership for EBP use [9] and nurse manager coaching and positive reinforcement [10] facilitate EBP uptake and use. However, there is a dearth of studies quantitatively investigating the relationship among nurse managers and unit climates for EBP. More recently, Fryer et al. [11] demonstrated that nurse managers’ affective commitment to a falls reduction program was significantly related (*p* < .001) to clinician support for the program and ultimately implementation success. Despite these examples, more rigorous research is needed to describe the leadership behaviors nurse managers strategically practice to support EBP implementation [4, 5, 11].

In addition to their unique position as leaders in healthcare organizations to provide support for EBP implementation, nurse managers adopt leadership styles and exhibit leadership behaviors and strategies that directly impact implementation [12, 13]. Birken and colleagues [1] contend that middle managers (nurse managers) influence implementation through diffusing information, synthesizing information, mediating between strategy and day-to-day activities, and promoting innovation implementation. Based upon these four domains, Engle and colleagues [14] identified 14 leadership strategies including use of communication mechanisms and styles, garnering staff involvement and buy-in, coaching staff through implementation processes, and providing resources and support.

Middle managers (e.g., nurse managers) are argued to contribute to implementation through their shaping of unit climates supportive of implementation [15, 16]. Klein and Sorra’s theory of innovation implementation contends that implementation climate, as a shared perception of members within a practice setting, is a predictor of implementation effectiveness and innovation uptake by users [17]. Weiner et al. note numerous challenges related to studying implementation climate, including alignment of the level of theory with the level of measurement and analysis [18]. Climate is argued to be a multilevel construct which can be measured at the individual level and unit and/or organizational level [18, 19]. Much of the climate literature focuses on organizational climate, which is an aggregate of individual perceptions emerging from interactions people have with each other [20]. This paper is primarily concerned with the relationship between an individual’s perception of their work environment and their perception of the unit’s leadership regarding implementation. Therefore, implementation climate and leadership were conceptualized, measured, and analyzed as individual level constructs.

Climate has been argued to be an important construct to implementation science because it influences implementation outcomes [17]. Using a mixed-methods approach, Damschroder and colleagues demonstrated the relationship of implementation climate to effectiveness of implementing a weight management program at Veterans Affairs medical centers [21]. Other focused climates have been studied more extensively, such as safety climate, and provided evidence for the relationship of climate and outcomes [22]. Considerable work is needed to more comprehensively understand implementation climate, identify its antecedents, and evaluate its influence on implementation of EBP and outcomes.

Implementation climates are believed to be strategically embedded and maintained by leaders [23, 24]. Leadership is a critical antecedent to climate [25–27] but has received very little empirical attention [22, 25]. Identifying and describing the associations among leadership and implementation climates is important for informing work to improve the context for implementation, which may ultimately improve implementation effectiveness. In mental health settings, Aarons and colleagues found positive associations among clinician ratings of middle managers’ leadership behaviors for implementation and organizational climate for implementation [28]. However, no published studies have examined these relationships in acute care nursing units with nurse managers. For this reason, the aim of this study was to examine the contributions of nurse manager EBP leadership behaviors and nurse manager EBP competencies in explaining climates for EBP implementation in adult medical-surgical units.

### Conceptual model

**Fig. 1:**
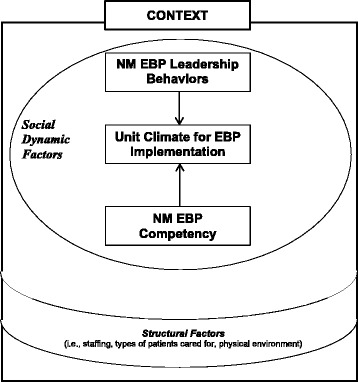
Conceptual model. NM nurse manager, EBP evidence-based practice

This study used a conceptual model derived from the Promoting Action on Research Implementation in Health Services (PARIHS) framework [29], which defines successful implementation as a function of the interactions among evidence, context, and facilitation [30]. Implementation of EBP occurs within widely diversified practice environments or contexts. For the purposes of this study, context includes both structural and social dynamic factors. Structural context includes key characteristics of the healthcare setting, such as staffing, unit size, and the types of patients cared for in the unit. Social dynamic context includes the roles, relationships, and dynamics among individuals and groups within a unit. In this study, we examine the social dynamic factors of unit climate, nurse manager leadership behaviors, and nurse manager competencies in EBP. The conceptual model for this study delineates the relationships among these three concepts (Fig. 1).

## Methods

### Design

A multi-site, multi-unit cross-sectional design was used to address the study aim. The study was conducted from 2016 to 2017 after obtaining approval from the University of Michigan Institutional Review Board and the ethics review boards at each participating hospital.

### Setting

The study was conducted in 24 units nested within seven acute care community hospitals in the Midwest and Northeastern USA. Hospitals were recruited through the National Nursing Practice Network, a network of over 100 community and academic hospitals representing 32 states in the USA. Inclusion criteria for study units from each hospital were (1) cared for patients ≥ 21 years of age; (2) designated as a medical, surgical, medical-surgical, or specialty unit (e.g., oncology, orthopedics, cardiac step-down unit); and (3) had a nurse manager who met inclusion criteria. Mother-baby, pediatric, neonatal, psychiatric, and critical care/intensive care units were excluded. For hospitals with more than one eligible unit managed by the same manager, one unit was randomly selected.

### Participants

The participants for this study were (1) nurse managers of the study units and (2) staff nurses caring for patients on the study units.

#### Nurse managers

A nurse manager was defined as a registered nurse who oversees unit-level operations in a hospital and is responsible for care delivered by clinical staff. Managers of nursing units have various titles such as nurse manager, unit director, and clinical coordinator. Inclusion criteria for nurse managers were (1) licensed as a registered nurse, (2) has responsibility and accountability for unit-level operations, (3) not serving in an interim role, and (4) is direct supervisor of nursing staff on the study unit. Senior nurse leaders held executive positions involving responsibility at that organizational level for operational activities related to healthcare delivery (e.g., Chief Nursing Officer). Twenty-four nurse managers were invited to participate.

#### Staff nurses

Staff nurse was defined as a licensed registered nurse providing direct patient care on a study unit. Inclusion criteria for staff nurses were (1) licensed as a registered nurse, (2) worked a minimum of .40 full-time equivalents, (3) provided direct patient care, and (4) was designated as staff on the study unit. Those who worked less than .40 full-time equivalents and were designated as contingency/agency staff or floats among units (float pool) were excluded. Thirty eligible staff nurses per unit were randomly selected to receive invitations to participate. For study units with < 30 eligible staff nurses (*n* = 13 units), all eligible were invited. The total sample size of staff nurses invited to participate was 553. A sample size of 277 staff nurse responses was needed to provide 80% power [31] in detecting a significantly small effect size (*d* = .04) with alpha of .05 using two-tailed tests, based on preliminary findings from our prior pilot study (unpublished).

### Study variables and measures

#### Dependent variable

Unit climate for EBP implementation (hereafter as “unit climate”) was defined as an individual’s perception of what is expected, rewarded, supported, and recognized regarding EBP implementation in the unit [20, 32]. Unit climate was measured using the 18-item *Implementation Climate Scale* (ICS), which measures the extent to which an employee perceives their unit to prioritize and value EBP based on six domains: (1) the unit’s focus on EBP, (2) educational support available for EBP, (3) recognizing staff for using EBP, (4) rewarding staff for using EBP, (5) selecting/hiring staff who value or use EBP, and (6) selecting/hiring staff open to innovation [32]. Respondents select their level of agreement with each item using a Likert scale from 0 to 4 (0 = not at all, 1 = slight extent, 2 = moderate extent, 3 = great extent, 4 = very great extent). Staff nurses completed this scale. The ICS total score is calculated for each participating nurse by summing scores for each item and dividing by 18. Internal consistency reliability and construct validity (exploratory and confirmatory factor analysis) have been demonstrated in mental health settings [32].

#### Independent variables

Nurse manager EBP competency (hereafter as “managerial competency”) was defined as a nurse manager’s expected level of purposeful performance regarding use of evidence to improve care delivery resulting from the integration of knowledge, skills, abilities, and judgment about EBP. It was measured using the 16-item *Nurse Manager EBP Competency Scale* (NM-EBPC) [33]. Nurse managers indicate their self-perceived level for each EBP competency using a Likert scale from 0 to 3 (0 = not competent, 1 = somewhat competent, 2 = fully competent, and 3 = expertly competent). Nurse managers completed this scale. The EBP competency scale total score is calculated by summing the scores on each item and dividing by 16. The NM-EBPC scale has demonstrated content validity and internal consistency reliability [33].

Nurse manager leadership behaviors for EBP implementation (hereafter as “unit leadership”) was defined as the specific leadership behaviors enacted by nurse managers to facilitate EBP implementation and foster an EBP climate on their unit(s) [12]. Unit leadership was measured using the 12-item *Implementation Leadership Scale* (ILS), which measures staff nurses’ perceptions of their nurse manager’s leadership behaviors supportive of EBP implementation in four domains: (1) proactive leadership, (2) knowledgeable leadership, (3) supportive leadership, and (4) perseverant leadership [12]. Respondents indicate their agreement with each item using a 0–4 Likert scale (0 = not at all; 4 = very great extent). This scale was completed by staff nurses. The total score is calculated by summing scores for each of the 12 items and dividing by 12. The ILS has demonstrated reliability and validity, including confirmatory factor analysis in mental health settings [12, 34].

#### Participant demographics

Demographic data included age in years, gender (male, female), race (Caucasian, other), shift (staff nurses only: days, evenings, nights, rotating), education level (diploma, associate, bachelor, master), years of experience as a registered nurse, years of experience as a nurse manager, and years of experience for both staff nurses and nurse managers in their current hospital and current unit. Years of experience on current unit and education level were included as confounding variables in all analyses.

#### Hospital and unit characteristics

The following data were collected on the hospitals: size (< 100 beds, 100–300 beds, > 300 beds), ownership type (private nonprofit, private for-profit, church affiliated), location (urban, rural), Magnet® designation (current, expired, or no designation), average daily hospital census, and average case mix index. Unit-level characteristics included average unit bed capacity, average daily unit census, average patient age, average skill mix (% registered nurse to other), average registered nurse hours per patient day, and clinical nurse specialist hours per week [none (0 h), part-time (1–39 h), full-time (40 h)].

### Data collection procedures

Study sites provided letters of intent to participate and identified site coordinators to facilitate data collection using a detailed data collection manual developed and tailored to each site. Site coordinators identified eligible study units and nurse managers‚ provided blinded lists of eligible staff nurses for random selection‚ and assisted with the distribution of survey questionnaires. Site coordinators were trained to the manual during 60–90 min teleconferences. Following training, any additional questions were addressed via email or phone. Site coordinators provided hospital and unit characteristic data using electronic data collection forms provided by the research team. (See Additional file 1 for the data collection timeline.)

Survey data were collected electronically from nurse managers and staff nurses using Qualtrics®, an online data collection software package [35]. Nurse managers were invited via email to complete a questionnaire that included the Nurse Manager EBP Competency Scale and demographic items. Staff nurses were invited via email to complete questionnaires inclusive of the Implementation Leadership and Implementation Climate scales, along with demographic items. Completion and return of the questionnaire signified consent to participate. Those not returning questionnaires within 1 week were sent an email reminder, with similar reminders sent each week, up to 1 month, as needed. Participants completing the questionnaire were offered an opportunity to voluntarily enter a lottery drawing for a chance to win a $100 cash gift card available to each unit.

### Statistical analysis

Data were analyzed using R version 3.1.2 [36]. Missing values were examined to identify potential missing patterns and determine a multiple imputation technique [37]. Staff nurse responses from units in which the nurse manager did not return the questionnaire were excluded from analyses. Missing confounding variables (including education and years of experience as registered nurse on current unit) were calculated with sequential regression multiple imputation [38]. Transformations were applied as needed to ensure normality and address homoscedasticity. Years of experience as a licensed registered nurse on the current unit were log-transformed. Internal consistency of the instruments was evaluated using Cronbach’s alpha. Significance was set at *p* < .05 for all analyses.

Contributions of unit leadership and managerial competency to unit climate were determined by *R*^2^ estimated from multilevel models using maximum likelihood [39]. For education level, diploma was used as reference category. To account for the nested structure of the data, analyses included random intercepts for (1) hospitals and (2) units within hospitals. Four models were computed with unit climate (individual level) as the dependent variable: (1) a model including the confounding variables and random effects, (2) a model adding the ILS scale to model 1, (3) a model adding the NM-EBPC scale to model 1, and (4) finally, a model adding both the ILS and NM-EBPC scales to model 1. The unique variance in unit climate explained by each added predictor(s) was calculated as 1 (residual variance with predictor/residual variance without predictor). Model fit was evaluated by comparing the Akaike information criterion (AIC) using the log-likelihood ratio test and marginal and conditional *R*^2^ [40].

## Results

### Hospital and unit characteristics

Hospital characteristics are described in Table 1. All seven hospitals were represented in the final sample, with roughly equal representation of small (< 100 beds; *N* = 3), medium (100–300 beds; *N* = 2), and large hospitals (> 300 beds; *N* = 2). Most hospitals were nonprofit (*n* = 6) and did not have current Magnet® designation (*n* = 5). Twenty-two of the 24 units were included in the analysis. Units varied in size (range 9–45 beds) and cared primarily for patients > 60 years of age (see Table 2).

**Table 1:** Hospital characteristics (*N* = 7)

	Number	Percent
Hospital size^a^		
< 100 beds	3	42.8
100–300 beds	2	28.6
> 300 beds	2	28.6
Hospital type (can be 1 or more)		
Private/not for profit	6	85.7
Private/for profit	1	14.3
Church affiliated	4	57.1
Urban	3	42.8
Rural	4	57.1
Magnet designation		
Current	2	28.6
Expired/no designation	5	71.4
	Mean	SD
Average daily hospital census^a^	132.49	138.44
Average case mix index^a^	1.41	0.40

^a^Represents average over 6 months

**Table 2:** Unit characteristics (*N* = 22)

	Mean	SD
Unit bed capacity^a^	24.58	9.76
Average daily unit census^a^	17.20	9.28
Average patient age^a^	64.18	5.44
Average skill mix^a^ (% RN to other)	59.00	10.00
Average RN HPPD^a^	7.27	1.55
	*n*	%
Clinical nurse specialist		
None (0 h)	9	40.9
Part-time (1–39 h)	9	40.9
Full-time (40 h)	4	18.2

*RN* registered nurse, *HPPD* hours per patient day

^a^Represents average over 3 months

### Participants

Twenty-two nurse managers of the 24 invited completed the NM-EBPC scale for a 91.7% response rate, which is higher than a previous study using this scale (63.8%) [33]. And, 287 staff nurses completed questionnaires for a 51.9% response rate, which is a similar response rate to a recent implementation study using a similar design and sample (47%) [41]. Twenty-three staff nurse observations were list-wise deleted because the nurse manager of their unit did not complete the NM-EBPC scale. Missing data on the ILS, ICS, and NM-EBPC scales were sparse and did not show any perceptible patterns. No individual item was missed more than four times and no respondent had missing information for more than two items. Therefore, missing items did not impede calculation of total scale scores for each respondent. We subsequently imputed missing data for education and/or years of experience as a registered nurse (*n* = 26 observations). This resulted in a final sample of 264 staff nurse observations, just under the estimated sample size needed based on the power analysis. Demographic characteristics of nurse managers and of staff nurses are described in Table 3.

**Table 3:** Participant demographics by role

	Nurse manager (*n* = 22)		Staff nurse (*n* = 264)	
	Mean	SD	Mean	SD
Age in years	41.76	6.67	35.44	11.94
Years as RN	15.64	6.06	8.28	10.1
Years as NM	3.91	2.56	NA	
Years in role in current hospital	3.95	2.61	5.85	8.14
Years in role in current unit	3.05	2.46	5.28	7.62
	*n*	%	*n*	%
Gender				
Female	20	90.9	236	89.4
Male	2	9.1	12	4.5
Prefer not to respond/missing			16	6.1
Race				
Caucasian	19	86.4	234	88.6
Other	3	13.6	16	6.1
Prefer not to respond/missing			14	5.3
Shift				
Days			102	38.6
Evenings	NA		10	3.8
Nights			68	25.8
Rotate			80	30.3
Missing			4	1.5
Education				
Diploma			7	2.7
Associates	3	13.6	87	33.0
Bachelors	12	54.6	155	58.6
Masters	7	31.8	7	2.7
Missing			8	3.0

### Scale reliabilities and descriptive statistics

The NM-EBPC scale demonstrated high internal consistency reliability (Cronbach’s alpha = .93; *N* = 22). The average score was 1.62 (*SD* = 0.50), indicating that nurse managers felt between “somewhat competent” (score of 1) and “fully competent” (score of 2) in EBP. Staff nurse responses to the ILS and ICS scales also demonstrated high internal reliabilities (Cronbach’s alpha for ILS = .97; ICS = .94; *N* = 264). The average ILS and ICS scores (0 to 4 range) were 2.84 (*SD* = 0.77) and 2.23 (*SD* = 0.75), respectively. In other words, staff nurses perceived their nurse managers to demonstrate leadership behaviors for EBP on average between a “moderate” and a “great extent.” Similarly, unit climates for EBP were perceived to be on average between a “moderate” and a “great extent.”

### Multilevel model results

Results of multilevel modeling are described in Table 4. The variance accounted for by the random intercepts (hospitals and units in hospitals) was significant in all models (*p* < .001). Model 1, and the other models as well, indicated that years of experience as a registered nurse in the current unit was significant [*b* = − 0.14 (models 1 and 3) and − 0.08 (models 2 and 4); *p* < .05], while education level (with diploma as the reference level) was not significant. In model 2, ILS scores explained 45.2% unique variance in unit climate and was significant (*b* = 0.65, *p* < .001), while, NM-EBPC total scores (model 3) explained less than 1% and was not significant (*b* = − 0.24, *p* = .142). The fit statistics (including AIC, marginal *R*^2^, and conditional *R*^2^) suggest that models 2 and 4 are best fit to the data. The log-likelihood ratio test comparing model 2 (with only ILS scores added) to model 4 (with both ILS scores and NM-EBPC scores) was significant [*x*^2^ (1) = 7.5024, *p* = .006], favoring model 4 as the best fit.

**Table 4:** Summary of multilevel models explaining unit Implementation Climate (ICS scores) (*N* = 264)

	Model 1 (control variables only)			Model 2 (model 1 + ILS)			Model 3 (model 1 + NM-EBPC)			Model 4 (model 1 + ILS + NM-EBPC)		
	*b*	SE	*p*	*b*	SE	*p*	*b*	SE	*p*	*b*	SE	*p*
Control variables												
Education	NS			NS			NS			NS		
Log years of experience as RN on unit	− 0.14	0.04	.001	− 0.08	0.03	.017	− 0.14	0.04	.002	− 0.08	0.03	.008
Unit leadership (ILS scores)				0.65	0.04	< .001				0.65	0.04	< .001
Managerial competency (NM-EBPC scores)							− 0.24	0.16	.142	− 0.25	0.08	.008
Unique variance explained by added predictor(s)				.452			.001			.452		
Fit statistics												
AIC	564.7			400.1			564.4			394.6		
Marginal *R*^2^	.044			.507			.065			.524		
Conditional *R*^2^	.253			.578			.251			.574		

Note: All models included random intercepts for hospital and unit within hospital

In model 4, ILS scores had a significant relationship with units’ implementation climate (*b* = 0.65, *p* < .001). NM-EBPC scores had a statistically significant relationship with implementation climate (*b* = − 0.25, *p* = .008) but accounted for only 0.001 variance in unit climate. The confounding effect of log years of experience as a registered nurse, although small, had a significant relationship with ICS total score (*b* = − 0.08, *p* = .008). Model 4 accounted for over 50% of variance in unit climate (marginal *R*^2^ = 0.524; conditional *R*^2^ = .574). Units within hospitals accounted for 3.13% of variance, and hospitals accounted for 7.42% variance in unit climate. There were no indications of problems with homoscedasticity or deviations from normality.

## Discussion

The purpose of this study was to investigate the contributions of unit leadership (behaviors and competencies) in explaining unit climate for EBP implementation. Unit leadership of nurse managers was associated with unit implementation climate, indicating that individual staff nurse perceptions of their nurse manager’s leadership behaviors were associated with their perceptions of unit implementation climates. This finding supports unit level findings in mental health facilities [28] and provides further evidence of the relationship between middle manager leadership and implementation of EBP [1, 2]. As patient care leaders within units, nurse managers have responsibility for the patient care delivered by their staff. Consequently, it is imperative that they provide leadership oversight of evidence-based implementation efforts, motivate their staff to adopt evidence-based practices, identify and secure appropriate support for EBP care, and communicate expectations for delivery and implementation of EBP. These behaviors and actions are strategic mechanisms middle managers may use to embed and facilitate unit climates more conducive to EBP implementation, routine use, and long-term sustainability [16].

Although nurse manager EBP competency was statistically significant, it explained very little model variance (0.1%). In a preceding pilot study (*N* = 33; unpublished), nurse manager EBP competency was correlated with staff perceptions of their unit climate for EBP implementation (Pearson’s *r* = .389, *p* = .025). In the present study, nurse manager leadership behaviors explained much more variance than EBP competency. Leadership behaviors are specific actions (e.g., establishing a plan to facilitate EBP implementation) related to facilitating a unit climate supportive of EBP implementation, whereas EBP competencies (e.g., can define EBP) may not directly affect unit climates. As a newly conceptualized and operationalized construct, more research is needed to further examine the effect and implications of nurse manager EBP competency in implementation.

On average, staff nurses with more years of experience rated their unit climates for EBP implementation lower than those with less experience. More years of experience in a unit lends to greater exposure to the implementation climate of the unit but may be associated with increased burnout and fatigue, possibly resulting in a more negative perception of their unit’s climate. Nurse managers who exemplify authentic leadership behaviors and create positive, empowering, and supportive work environments are key to reducing burnout and improving job satisfaction of nurses in their unit [41, 42]. Consequently, more work is needed to improve the leadership of nurse managers to improve unit climates and work environments. In addition, the significance of years of experience in the unit in explaining unit climate suggests that more studies are needed which examine relationships among social dynamic factors (e.g., unit leadership and climate) and clinician characteristics (e.g., years of experience and education), unit characteristics (e.g., staffing), and hospital characteristics (e.g., Magnet status and academic versus community).

Investigating the role of context, including leadership and climate, in implementation and sustainability of EBP is a high priority [43, 44]. Units with climates more favorable to EBP implementation and with nurse managers who exhibit higher levels of leadership behaviors for EBP may provide an ideal setting for implementation efforts. More studies are needed to investigate relationships among these social dynamic context factors at other levels with a hospital (e.g., unit, department, hospital). Climate is often studied at the unit level by aggregating participant responses. Interrater agreement and reliability within a unit is argued to be representative of climate strength, which has demonstrated direct and moderating effects on climate level and outcomes [22]. Future research should apply findings from this study to inform investigation of leadership and climate at the unit level. Additionally, studies are needed which examine interactions and relationships among idiosyncrasies in individual perceptions of EBP climate and leadership with ratings aggregated to the unit and organizational level, which may help us better understand the influence of leadership and climate strength in implementation [22].

Additional research is warranted to determine the relationships and influence of implementation leadership behaviors and climates on other outcomes (e.g., implementation success, EBP use) and, subsequently, develop interventions to improve them [13, 45]. Interventions addressing the development and maintenance of unit climates for EBP are likely to improve current and future EBP implementation and sustainability efforts [32]. These interventions should include nurse managers by (1) evaluating and addressing a nurse manager’s leadership for EBP and (2) delineating methods for rewarding and recognizing staff for EBP, supporting education of staff about EBP, and hiring staff with knowledge and skills in EBP. Additionally, future research is needed to further explicate the relationship of these social dynamic factors and examine them in other care settings (e.g., pediatrics, intensive care, ambulatory care, long-term care).

### Limitations

This is the first multi-site study to examine associations among nursing leadership and unit climate for EBP implementation in acute care. This study has numerous limitations. First, hospitals were conveniently selected; however, to mitigate this limitation and improve generalizability, different-sized hospitals representing different regions in the USA were recruited. In addition, to reduce bias resulting from hospitals selecting better performing units to participate, all eligible units were included, with one unit being randomly selected from units managed by the same nurse manager.

As a cross-sectional study, associations rather than causation between variables were examined. Results interpreted in light of common methods bias as responses to the ILS (independent variable) and ICS (dependent variable) were collected from the same sample, at the same time, and using the same method. Future studies should address this limitation in the study design. In addition, experimental studies should consider including these social dynamic context variables. Staff nurse and nurse manager responses were collected at one point in time and did not take into account EBP implementation efforts previously or currently in progress on the units. Units currently implementing EBPs may have performed better on some of the instruments due to increased attention to EBP implementation. Also, observing trends or stability in perceptions over time may have provided a more robust understanding of the social dynamic context for implementation.

### Implications for implementation science

Context for implementation is markedly dynamic and is an important challenge in the field of implementation [46]. Clinical interventions developed in efficacy and effectiveness trials often require adaptations to specific contexts [47]. In addition to developing implementation strategies that adapt specific interventions to a particular setting, interventions are needed which address context factors (e.g., leadership, climate). Such intervention work is being developed and tested in mental health agencies [48] and home-care nursing [49]. However, interventions are needed to address nurse managers of acute care nursing units. The empirical evidence supporting the association between nurse manager leadership and unit climate for implementation provides valuable knowledge to inform development of interventions aimed at improving nurse managerial leadership to embed and facilitate climates more conducive of implementation.

## Conclusions

Unit climates for implementation are related to nurse manager leadership behaviors, and yet, average values for unit leadership were modest at best. Future studies should consider using these measures to better explicate and address factors that foster or hinder EBP implementation. The development and testing of interventions targeting the leadership behaviors and competencies of nurse managers is essential to advance the science in this field and to improve acute care practice environments.

## Additional file

Additional file 1: Data Collection Timeline. (PDF 51 kb)

## Abbreviations

AIC: Akaike Information Criterion
EBP: Evidence-based practice
ICS: Implementation Climate Scale
ILS: Implementation Leadership Scale
NM-EBPC: Nurse Manager EBP Competency Scale
