# Supplementary material for: Associations among unit leadership and unit climates for implementation in acute care: a cross-sectional study
Source: Implement Sci. 2018 Apr 25;13:62. doi: 10.1186/s13012-018-0753-6 (PMC5918552; doi:10.1186/s13012-018-0753-6)
Supplement: Supplementary file 1 — Data Collection Timeline. (PDF 51 kb) [file 13012_2018_753_MOESM1_ESM.pdf]

Associations among unit leadership and unit climates for implementation in acute care: a cross-sectional study

Clayton J. Shuman, PhD, RN (clayshu@umich.edu) et al.

Supplementary Material: Data Collection Timeline

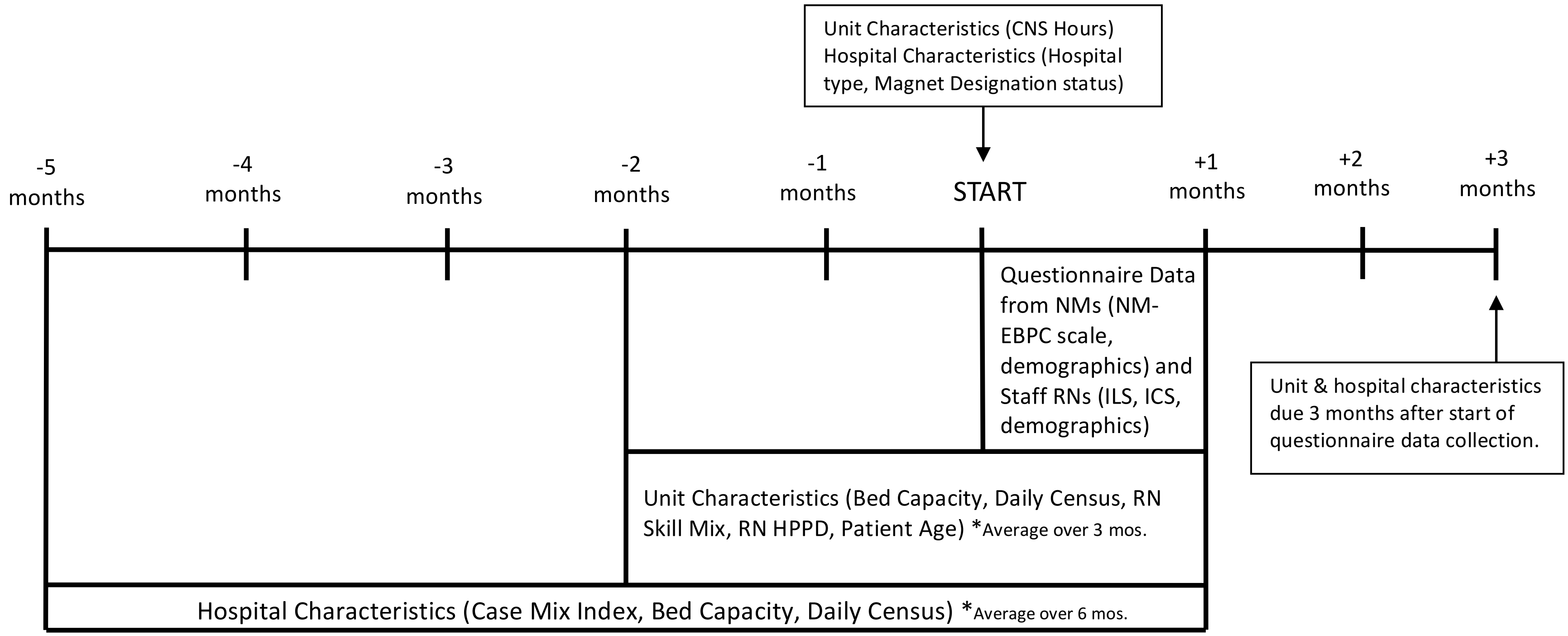

Note: CNS= clinical nurse specialist; NM= nurse manager; RN= registered nurse; NM-EBPC= *Nurse Manager EBP Competency Scale*; ILS= *Implementation Leadership Scale*; ICS= *Implementation Climate Scale*; HPPD= hours per patient day.
